# The dual effect of mesenchymal stem cells on tumour growth and tumour angiogenesis

**DOI:** 10.1186/scrt195

**Published:** 2013-04-29

**Authors:** Michelle Kéramidas, Florence de Fraipont, Anastassia Karageorgis, Anaïck Moisan, Virginie Persoons, Marie-Jeanne Richard, Jean-Luc Coll, Claire Rome

**Affiliations:** 1 Inserm U823, Institut Albert Bonniot, Université Joseph Fourier, Rond-Point de la Chantourne, Grenoble, 38706, France; 2 UM Biochimie des Cancers et Biothérapies, CHU de Grenoble, Institut de Biologie et Pathologie, Parvis Belledonne, Grenoble, 38043, France; 3 Inserm U836, Grenoble Institut des Neurosciences, Université Joseph Fourier, Chemin Fortuné Ferrini, Grenoble, 38706, France

## Abstract

**Introduction:**

Understanding the multiple biological functions played by human mesenchymal stem cells (hMSCs) as well as their development as therapeutics in regenerative medicine or in cancer treatment are major fields of research. Indeed, it has been established that hMSCs play a central role in the pathogenesis and progression of tumours, but their impact on tumour growth remains controversial.

**Methods:**

In this study, we investigated the influence of hMSCs on the growth of pre-established tumours. We engrafted nude mice with luciferase-positive mouse adenocarcinoma cells (TSA-Luc^+^) to obtain subcutaneous or lung tumours. When tumour presence was confirmed by non-invasive bioluminescence imaging, hMSCs were injected into the periphery of the SC tumours or delivered by systemic intravenous injection in mice bearing either SC tumours or lung metastasis.

**Results:**

Regardless of the tumour model and mode of hMSC injection, hMSC administration was always associated with decreased tumour growth due to an inhibition of tumour cell proliferation, likely resulting from deep modifications of the tumour angiogenesis. Indeed, we established that although hMSCs can induce the formation of new blood vessels in a non-tumoural cellulose sponge model in mice, they do not modify the overall amount of haemoglobin delivered into the SC tumours or lung metastasis. We observed that these tumour vessels were reduced in number but were longer.

**Conclusions:**

Our results suggest that hMSCs injection decreased solid tumour growth in mice and modified tumour vasculature, which confirms hMSCs could be interesting to use for the treatment of pre-established tumours.

## Introduction

Mesenchymal stem cells (MSCs), also referred to as stromal progenitor cells, reside in the adult bone marrow, are capable of self-renewal and can be quickly expanded *in vitro*. Under appropriate experimental conditions, MSCs can differentiate into a number of mesodermal cell lineages, including bone, cartilage, stroma, adipose tissue, connective tissue, muscle and tendon [1-3]. MSCs also have immunosuppressive properties that can be exploited for the treatment of autoimmune or graft-versus-host diseases [4].

Moreover, MSCs possess an innate tropism for sites of injury irrespective of tissue or organ type. MSCs have been shown to specifically home to tumour and metastasis sites in multiple types of cancers [5]. The tropism of MSCs for tumours is thought to be due to a similarity in the factors secreted by wounds and tumours, which has led to the hypothesis that tumours resemble chronic wounds or ‘wounds that never heal’ [6]. Thus, MSCs have been studied as potential anti-tumour cells and as vehicles for gene therapy [7].

However, the role of native MSCs inside the tumour microenvironment is unclear, and the relationship between MSCs and tumour cells is complicated; MSCs have been linked to contradictory effects on tumour growth [8-11]. This dual influence of MSCs is also present in the angiogenic process. MSCs are capable of inducing neoangiogenesis *in vitro*[12,13] and *in vivo*[14-16]. In addition, they can differentiate into endothelial cells [17,18] and/or pericytes [19]. In some tumour models, grafted MSCs were shown to settle into the tumour vessel walls, inducing a pro-angiogenic effect [20] and an increase in tumour growth [21]. In contrast, in other tumour models, MSCs have been reported to abrogate tumour growth by pushing endothelial cells to enter programmed cell death [22].

To clarify this issue, we studied the impact of human mesenchymal stem cells (hMSC) injection on pre-established tumours. We demonstrated that hMSC administration decreases tumour growth when injected systemically or directly in contact with the tumours, suggesting a systemic effect. This effect is clearly correlated with an inhibition of tumour cell proliferation. Furthermore, we established that although hMSCs induced physiological angiogenesis *in vivo*, these cells do not alter the overall pathological angiogenesis of the tumours. However, administration of hMSCs induced significant remodelling of the tumour vasculature. These observations suggest that tumour vasculature normalisation is induced by the presence of hMSCs.

## Materials and methods

### Cell culture

TSA-pGL3 is a cell line derived from the murine mammary adenocarcinoma TS/A-pc cell line comes from spontaneous mammary tumour cells [23] that has been stably transfected with the pGL3-luciferase reporter gene (Promega, Charbonnières, France). Cells were cultured at 37°C in a humidified 5% CO_2_ incubator in RPMI 1640 media supplemented with 1% glutamine, 10% foetal bovine serum, 50 units/ml penicillin, 50 μg/mL streptomycin, 25 μM β-mercaptoethanol and 700 μg/ml Geneticin™ (G418 sulphate; Gibco, Paisley, UK).

hMSCs were isolated from bone marrow aspirates from three healthy donors, each of whom gave informed consent. All the isolation and culture procedures were conducted in the authorized cell therapy unit (Biotherapy Team of the General Clinical Research Center, French Health Minister agreement TCG/04/0/008/AA) at the Grenoble University Hospital. The cells were cultured at 37°C in a humidified atmosphere containing 5% CO_2_ according to a previously described method [24]. Briefly, hMSCs were selected by plastic adhesion and cultured in Minimum Essential Eagle Medium alpha (MEMα) supplemented with 100 μg/mL penicillin, 100 μg/mL streptomycin, and 10% foetal calf serum (FCS) (all reagents from Invitrogen, Cergy Pontoise, France). All the hMSCs used were phenotyped by FACS analysis and their functionality was tested (colony-forming units and *in vitro* differentiation in adipocyte and osteoblast [24]) (data not shown).

### Cell implantation in mice

**Figure 1:**
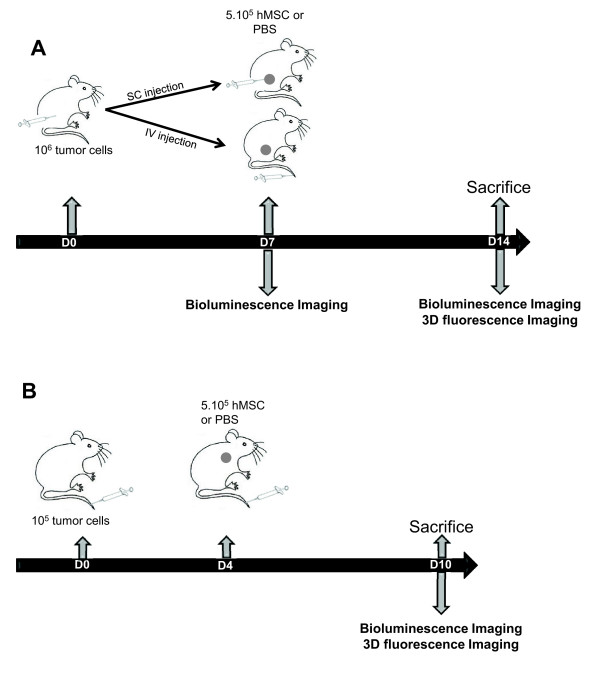
**Lung metastasis and subcutaneous tumour models. (A)** Subcutaneous tumour model. At day 0, 10^6^ TSA-pGL3 cells/200 μL were SC injected (n = 15). At day 7, bioluminescence imaging was performed for the three homogeneous groups. The control group was IV injected with 200 μL of PBS, the hMSCs SC group was peritumourally injected with 5 × 10^5^ cells/200 μL PBS and the hMSCs IV group was IV injected with 5 × 10^5^ cells/200 μL PBS. At day 14, bioluminescence and three-dimensional fluorescence imaging were performed. The mice were euthanized, their Hb contents were measured, and the tumours were frozen for immunohistological studies. **(B)** Lung metastasis model. At day 0, 10^5^ TSA-pGL3/200 μL PBS cells were IV injected (n = 8). At day 4, 200 μL of PBS (control) or hMSCs (5 × 10^5^ cells/200 μL PBS) were IV injected. At day 10, bioluminescence and three-dimensional fluorescence imaging were performed. The mice were euthanized, and their Hb contents were measured. Hb, haemoglobin; hMSC, human mesenchymal stem cell; IV, intravenous; PBS, phosphate-buffered saline; SC, subcutaneous; SD, standard deviation; TSA-pGL3, TS/A-pc mouse adenocarcinoma cell line stably transfected with the luciferase reporter gene.

All animal experiments were conducted in adherence to the Principles of Laboratory Animal Care (National Institutes of Health publication no. 86–23, revised 1985) and approved by the regional ethics committee (Reference number of animal experiments: 96_IAB-U823 MK-09 and 97_IAB-U823 MK-08; Comité d’éthique en expérimentation animale de Grenoble: Com-Eth, amended by the Comité National de Réflexion Ethique sur l’Expérimentation Animale (No.12)). Female athymic NMRI nude mice, purchased from Janvier (Le Genest Saint Isle, France) at 6 to 8 weeks of age, were maintained under specific pathogen-free conditions. We choose to inject the TSA-pGL3 cell line (1) subcutaneously because it is an easy model to follow and visualize and (2) intravenously to mimic a more physiological lung metastasis model [25] (Figure 1). The subcutaneous (SC) injection of 10^6^ TSA-pGL3 cells suspended in 200 μL of PBS into the right flank of mice resulted in the formation of 6 to 8 mm-diameter tumours after one week. Intravenous (IV) injection of 10^5^ TSA-pGL3 cells suspended in 200 μL of PBS into the tail vein resulted in tumour lung nodule formation at day 4. At these times, animals were randomised into groups (*n* = 5 per group), and 5 × 10^5^ hMSCs were injected subcutaneously at the tumour site or intravenously.

### Mouse subcutaneous sponge angiogenesis assay

Cellspon cellulose sponges (thickness 2 mm, diameter 10 mm, Cellomeda; Turku, Finland) were implanted under the skin of NMRI nude mice [26]. Operations were performed under general anaesthesia induced by intraperitoneal injections of Domitor™ (Pfizer, Orsay, France) and Imalgene™ (Merial, Lyon, France). The sponges were hydrated with 50 μL of PBS or FGF-2 (200 ng/50 μL; FGF-2: recombinant human FGF-basic, Eurobio Abcys S.A., Les Ullis, France), or 10^4^ hMSCs were deposited on the sponge surface. Each group contained five mice. For the FGF-2 group, FGF-2 (50 μL) was again injected into the sponges through the skin on day 2 and 3. At 7 days after implantation, the mice were anesthetised, and the sponges were rapidly excised and photographed. Each sponge was then homogenised in 1 mL RIPA lysis buffer with protease inhibitors, and the supernatants were subjected to haemoglobin quantification using Drabkin’s reagent (Sigma-Aldrich, Saint-Quentin Fallavier, France), expressed as mg/ml.

### Bioluminescence and three-dimensional fluorescence *in vivo* imaging

All imaging was performed under inhalational anaesthesia (3% isoflurane) and administered to a free breathing mouse using a nose cone. For bioluminescence imaging, mice received an intraperitoneal injection of D-luciferin potassium salt dissolved in sterile phosphate-buffered serum (150 mg/kg) 5 min before imaging (ORCAII-BT-512G, Hamamatsu Photonics, Massy, France), as described previously by Jin *et al*. [27,28]. Semi-quantitative data were obtained from the bioluminescence images by drawing regions of interest on the area to be quantified. Images were acquired as 16-bit TIFF files, which can provide a dynamic of up to 65,535 grey levels. Measurement of the bioluminescence intensities (expressed as the number of relative light units (RLU) per pixel per second for each region of interest (ROI)), were performed using the Wasabi software (Hamamatsu). The colour scale values displayed by the software are adjusted to the indicated maximum values. For the lung metastasis model, because the signal is too low before day 5, bioluminescence signals were measured 10 days after tumour cell injection and quantified at the end of the experiment to determine the effects of hMSCs on tumour growth. For subcutaneous tumours, bioluminescence signals were followed from day 7 (before hMSC injection) to day 14 after tumour cell implantation, and tumour growth rate was calculated as the ratio of bioluminescence signal between D14 and D7 (D14/D7).

For three-dimensional fluorescence imaging, 200 μl Alexa700-RAFT-c(−RGDfK-)_4_ (50 μM; Angiostamp™; Fluoptics, Grenoble, France), which targets the integrin α_v_β_3_, was injected intravenously through the tail vein of each mouse. Three-dimensional fluorescence acquisition and quantification were performed 16 h post-injection for the tumour models and 3 h for the sponge model with the continuous-wave fluorescence-enhanced diffuse optical tomography system previously described by Koenig *et al*. [29,30]. fDOT consists of a 690-nm laser source, a CCD camera and a set of filters. The light source is a 35-mW compact laser diode (Power Technology, Little Rock, AR, USA) equipped with a bandpass interference filter (685AF30OD6; Melles Griot, Albuquerque, NM, USA). The emitted fluorescence is filtered by two 700-nm high-pass colored glass filters (RG9 OD5; Schott, Mainz, Germany) placed in front of a NIR sensitive CCD camera (Hamamatsu Photonics K.K., Japan) mounted with a f/15-mm objective (Schneider Kreutznach, Bad Kreuznach, Germany). The excitation sources described a regular 11 × 11, 2-mm spaced grid (2 × 2 cm^2^ field of view) over the region of the mouse where the tumour is implanted. Two scans were successively performed for diffusion and fluorescence. The exposure time was automatically computed at each laser position to use the entire dynamic range of the camera. The two stacks of diffusion and fluorescence images were analysed by the reconstruction algorithm to generate a three-dimensional image [31]. The fDOT principle lies in the ability to both reconstruct fluorescence even in highly heterogeneous-attenuating media and handle complex geometries. The results are presented as a three-dimensional view of the reconstructed area. The reconstructed area is a volume meshed with a 2-mm sample rate in the x and y directions and 1 mm in the z direction (depth) that yields a size of approximately 8 x 10 x 15 voxels and may vary slightly depending on animal thickness. The cross-sections are presented from bottom to top for z = 0 (ventral side) to z = 15 (dorsal side). The superimposition of the reconstructed volumes viewed as a smooth interpolation perspective and positioned on top of the white-light image of the animal allowed for the generation of the final image. The procedure time on a 3-GHz Intel Xeon was 10 min to reconstruct the fluorescence distribution. Each fluorescence reconstruction is presented with the same color scale to allow for visual comparison. The scale is provided in arbitrary units because the tomography produces relative values unless a standard calibration has been performed Keramidas *et al*. [32].

### CD31, αSMA and Ki67 immunohistochemistry

Frozen sections (8 μm) from subcutaneous tumours were fixed in acetone for 10 min. Sections were then washed three times for 5 min each in Tris-buffered saline containing 0.1% Tween-20 and endogenous peroxidases were blocked with 0.1% H_2_O_2_ in methanol for 20 min. Sections were then sequentially incubated for 1 h with a rat monoclonal anti-CD31 antibody (MEC13.3; 1:500; BD Pharmingen, Pont de Claix, France), rabbit anti-Ki67 (1:100; Abcam, Paris, France) or rabbit anti-alpha smooth actin antibody (αSMA; 1:200; Abcam, Paris, France) and for 1 h with goat anti-rat (1:500; Cell Signaling Technology, Danvers, MA, USA) or goat anti-rabbit (1:200; Dako, San Antonio, TX, USA) secondary antibodies, as appropriate. Peroxidase activity was revealed using diaminobenzidine tetrachloride as a chromogen (Dako; San Antonio, TX, USA). Sections were counterstained with haematoxylin and mounted.

Immunohistochemical staining against the endothelial marker CD31 was followed by observation under a low magnification scope (100×) for five fields of view of each tumour (five tumours by condition). Then, vessel length and quantity were measured in each of these areas using ImageJ software [33]. All counts were performed in a blinded manner.

After immunohistochemical staining against Ki67 and αSMA, slides were observed under a high magnification microscope (200×). For Ki67, six to nine areas were photographed for each tumour. For αSMA, two to nine areas were photographed for each tumour. These photographs were analysed using the ImmunoRatio plug-in from ImageJ software [33]. The Ki67 index and the percentage of αSMA positive cells were evaluated in a blinded manner and calculated as the number of positive cells divided by all tumour cells in one field.

### RNA isolation and RT-PCR

Total RNA was extracted from tumours and organs with TRIzol™ (Invitrogen, Cergy Pontoise, France). The total RNA was quantified using a Nanodrop ND-2000 instrument (NanoDrop Technologies, Thermo Fisher Scientific, Wilmington, DE) and reverse transcription was performed on 1 μg total RNA with SuperScript III™ RNaseH reverse transcriptase (Invitrogen, Cergy Pontoise, France) under the conditions recommended by the manufacturers. Before PCR, quantities of cDNA samples were adjusted to yield equal amplifications of the mRNA encoding the housekeeping gene GAPDH (Forward: ACTCCACTCACGGCAAATTC, Reverse: TCTCCATGGTGGTGAAGACA). PCR for GAPDH and human CD90 (Forward: CCAACTTCACCAGCAAATACAA, Reverse: ACTGTGACGTTCTGGGAGGA) were performed in a final volume of 25 μl containing 1 × PCR buffer, 2 mM MgCl_2_, 200 μM dNTPs, 400 nM each primer, 0.5 U Taq polymerase (GoTaq™ Hot Start polymerase; Promega, Charbonnières, France). The PCR conditions were: step 1, 95°C for 30 sec, step 2, 62°C for 30 sec, step 3, 72°C for 30 sec. To ensure semi-quantitative results in the RT-PCR assays, the number of PCR cycles was selected to be in the linear range of amplification for each set of primers (n = 30 for GAPDH and n = 35 for hCD90). PCR products were separated and visualised after electrophoresis on 2% agarose gels containing 1 μg/ml ethidium bromide.

### Statistical analyses

Statistical analyses were performed using StatView™ (SA, Cary, NC, USA). All results are expressed as the mean ± standard deviation. Comparisons between groups were performed using a two-tailed Student’s *t* test. Statistical significance was assumed when *P* <0.05.

## Results

### hMSCs derived from bone marrow inhibit tumour growth *in vivo*

**Figure 2:**
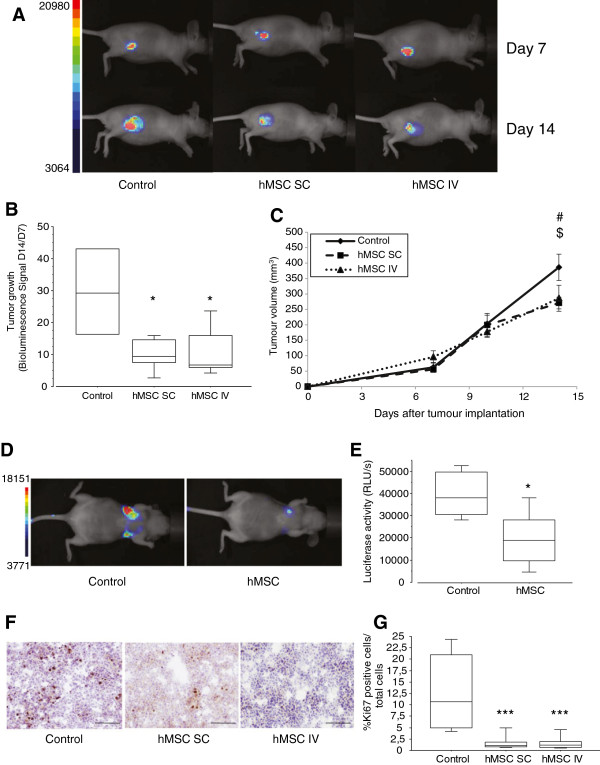
**Effects of hMSCs on tumour growth. (A)** Representative whole-body bioluminescence images of nude mice bearing SC tumours after injection with 10^6^ TSA-pGL3 cells before hMSC treatment (day 7) and treated with hMSCs (5 × 10^5^ cells; SC or IV injection; day 14) or PBS (control) (n = 4 to 5 mice/group; representative data from one of three different experiments are shown). **(B)** Box plots of tumour growth in each group. Tumour growth rate was calculated as the ratio of the bioluminescence imaging values obtained at days 14 and 7 (D14/D7) after tumour cell inoculation. The results are expressed as the mean ± SD. ^*^P <0.05. **(C)** The xenograft tumour volumes of the mice were determined at the indicated time points. hMSC were injected at day 7. Data are expressed as mean ± SD. ^#^ indicates *P* = 0.01 by Student's *t* test (control vs. hMSC SC); ^$^ indicates *P* = 0.07 by Student's *t* test (control vs. hMSC IV). **(D)** Representative whole-body bioluminescence images of nude mice bearing lung tumours after IV injection with 10^5^ TSA-pGL3 cells and hMSC treatment (5 × 10^5^ cells; IV injection; hMSCs) or PBS (control) (n = 4 mice/group). **(E)** Box plots of bioluminescence imaging results obtained at day 10 after tumour cell inoculation. The results are expressed as the mean ± SD. ^*^P <0.05. **(F)** Photographs of representative SC tumours stained with haematoxylin and a Ki67 antibody at day 14 and visualised at 200× magnification. **(G)** Box plots of Ki67^+^ proliferating tumour cells. The results are expressed as the mean ± SD (n = 6 to 9 photographs/tumour; five mice/group; ^***^P <0.0001). hMSC, human mesenchymal stem cell; IV, intravenous; PBS, phosphate-buffered saline; SC, subcutaneous; SD, standard deviation; TSA-pGL3, TS/A-pc mouse adenocarcinoma cell line stably transfected with the luciferase reporter gene.

Several reports have indicated that MSCs may influence tumour progression in different tumour types (reviewed by Klopp *et al*. [34]). In the present study, we investigated the effect of systematically or subcutaneously delivered hMSCs on TSA-pGL3 tumours (Figures 1 and 2). First, tumour cells were implanted SC and 7 days later, when tumours were macroscopically detectable, hMSCs were injected IV or SC in the periphery of the tumour (Figure 1A). In the second model, in the lung metastatic model, tumour cells were injected IV, followed by an IV injection of hMSCs 4 days later (Figure 1B). The injection of hMSCs alone did not result in tumour formation (data not shown). Tumour initiation and growth was followed and quantified by non-invasive bioluminescence imaging and calliper when possible (Figure 2A-E). In each tumour model, hMSC administration decreased the tumour growth rate and led to a delay in disease progression. Indeed, as shown in Figure 2B at day 14, bioluminescence rate (D14/D7) within subcutaneous tumours was lower when hMSCs were inoculated near the tumour site (control vs. hMSC SC: 2.88-fold; *P* = 0.03) or by the IV route (control vs. hMSC IV: 2.73-fold; *P* = 0.05). This also corresponded to a slight decrease in tumour volumes (Figure 2C). These results were confirmed by repeating the experiments using hMSCs from two other donors (data not shown).

In the lung metastasis model, at day 10 after IV inoculation with TSA-pGL3 cells, mice also injected with hMSCs exhibited a 2.03-fold (*P* = 0.0264) smaller bioluminescence signal than the control mice (Figure 2D-E).

**Figure 3:**
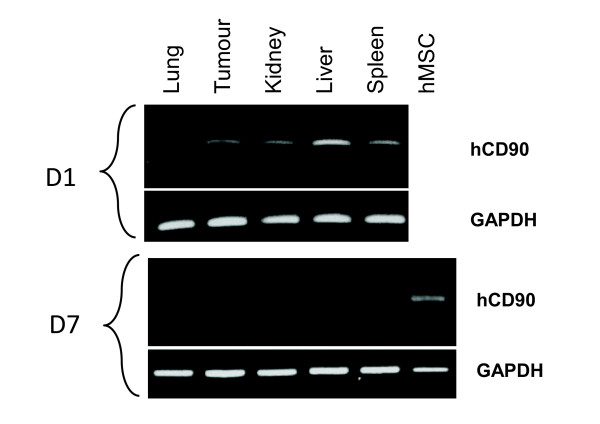
**Detection of hMSCs at different timepoints after IV injection.** Different tissues/organs were collected at various timepoints (day 1 (D1) and 7 (D7); n = 3) after the infusion of hMSCs. RNA was extracted from these tissues and analysed by RT-PCR. Human-specific CD90 (hCD90) and GAPDH mRNA expression were detected by electrophoresis in a 2% agarose gel (hCD90 product size: 139 bp, GAPDH product size: 170 bp). bp, base pair; hMSCs, human mesenchymal stem cells; IV, intravenous; RT-PCR, reverse-transcriptase polymerase chain reaction.

At day 14, animals were euthanized and the tumours were cryopreserved. Analyses of cell proliferation were performed by Ki67 labelling in sections from subcutaneous TSA-pGL3 (control) and TSA-pGL3 + hMSCs tumours (see Figure 2F-G). In the control tumours, 12.7 ± 7.6% of cells were Ki67 positive. The presence of hMSCs dramatically reduced the percentages of Ki67-positive cells, to 1.9 ± 1.9% and 1.7 ± 1.5%, when hMSCs were injected at the tumour periphery or IV, respectively (*P* <0.0001). The anti-proliferation effect was not significantly different when the hMSCs were injected systematically or near the tumour site, suggesting that the inhibitory effect of hMSCs on tumour cell growth could be indirect. Indeed, one day after hMSCs IV injection, human CD90 mRNA expression was detected by RT-PCR in different tissues and organs, including subcutaneous tumours (Figure 3), whereas the presence of the human CD90 mRNA was no longer detectable 7 days later.

### hMSCs stimulate *in vivo* angiogenesis

**Figure 4:**
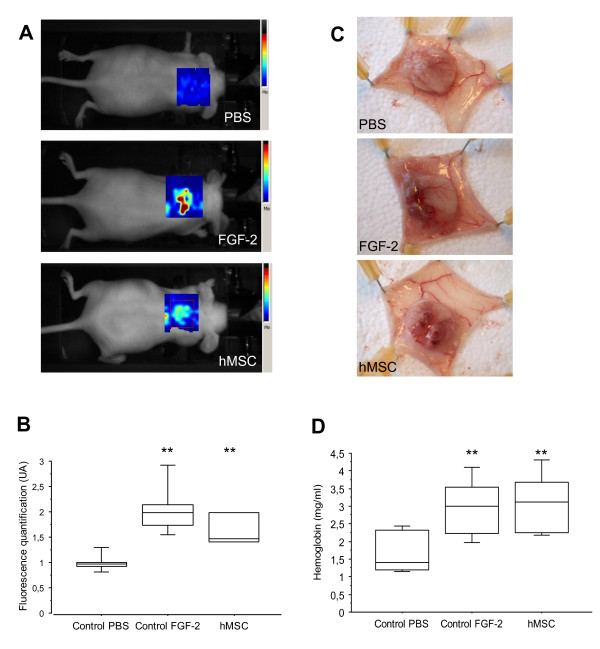
**hMSCs promote angiogenesis in an *in vivo *sponge model.** NMRI nude mice bearing a subcutaneous cellulose sponge treated with PBS (negative control) or FGF-2 (200 ng; positive control) or hMSCs (10^4^ cells) under the dorsal skin. **(A)** Representative three-dimensional fluorescence images of mice after IV injection of 50 μM Alexa700-labeled RAFT-c(−RGDfK-)_4_. **(B)** Box plot of fluorescence intensity, recorded as photons per pixel for a specified region of interest (ROI). The data are expressed as the mean ± SD (n = 3 to 6). **(C)** Animals were euthanised on day 7, and the sponges were photographed. **(D)** The amount of haemoglobin (Hb) was higher in the FGF-2- and hMSC-treated groups than in the PBS-treated group. The data are expressed as the mean ± SD of two experiments (12 mice in each group; ^**^P <0.0007). hMSC, human mesenchymal stem cell; IV, intravenous; PBS, phosphate-buffered saline; SC, subcutaneous; SD, standard deviation.

We then hypothesised that the influence of hMSCs on tumour growth was mediated by an indirect effect on tumour microenvironment and especially on the formation of blood vessels. We thus evaluated the capacity of hMSCs to induce angiogenesis using a mouse subcutaneous sponge assay [35] (Figure 4). When poorly angiogenic cellulose sponges are engrafted under the skin, very few blood vessels were observed seven days after their engraftment (PBS condition), and thus their haemoglobin (Hb) content after dissection was low (1.695 mg Hb/mL). In contrast, in hMSC- or FGF-2-treated sponge implants, a strong angiogenic response was observed, with a large invasion of the sponge by neo-formed blood vessels (Figure 4C).

This effect was quantified (1) by three-dimensional Alexa700-RAFT-c(−RGDfK-)_4_ fluorescence imaging (Figure 4A and 4B) and (2) by measuring the Hb content of the sponges (Figure 4D). The RGD-mediated fluorescence was 1.7-fold higher in sponges containing hMSCs compared to control sponges, suggesting an augmentation of the number of proliferative neo-endothelia known to overexpress the α_v_ß_3_ integrin. This pro-angiogenic activity of hMSCs was similar to that obtained after injection of FGF-2 (Figure 4B, *P* >0.05). Similar results were obtained when we quantified the Hb content in the sponges instead of using the RGD-based assay. Again, hMSC treatment was as pro-angiogenic as FGF-2 treatment (3.094 mg Hb/mL ± 0.816 for hMSCs and 2.939 mg Hb/mL ± 0.824 for FGF-2) (Figure 4D). These results confirm the *in vivo* pro-angiogenic potential of hMSCs.

### hMSC treatment induces profound tumour blood vessel reorganisation

To determine whether the influence of hMSCs on tumour growth could be related to the pro-angiogenic properties of the cells, we evaluated the vascularisation of the tumours after hMSC inoculation.

**Figure 5:**
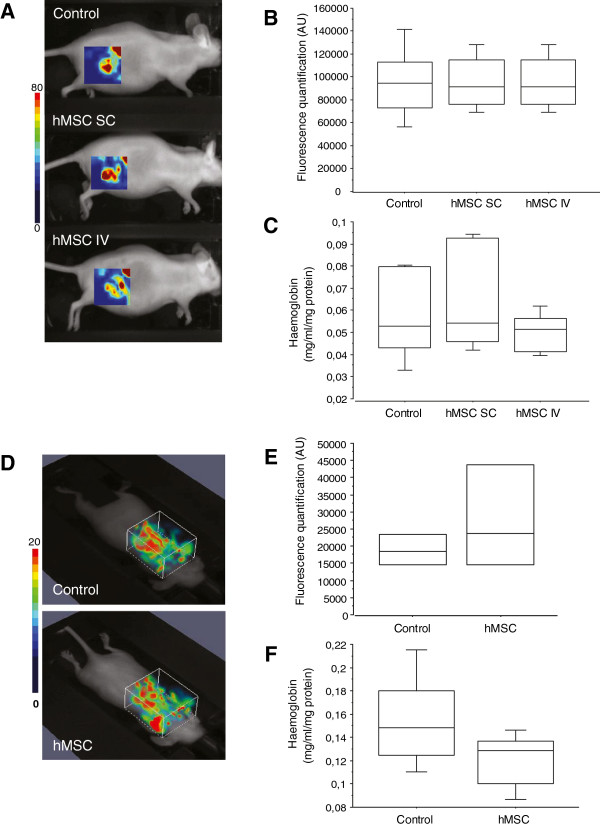
***In vivo *evaluation of tumour angiogenesis. (A** and **D)** Representative three-dimensional fluorescence images of mice bearing SC tumours (A) or lung tumours (D) treated or not with hMSCs, after IV injection of 50 μM Alexa700-labeled RAFT-c(−RGDfK-)_4_. **(B** and **E)** Box plot of fluorescence intensity, recorded as photons per pixel for a specified region of interest (ROI). The data are expressed as the mean ± SD (n = 4 to 5). **(C** and **F)** Amount of haemoglobin (Hb) quantified in subcutaneous (C) or lung (F) tumours. The data are expressed as the mean ± SD. hMSC, human mesenchymal stem cell; SC, subcutaneous; SD, standard deviation.

*In vivo* imaging of subcutaneous or lung tumours using Alexa700-RAFT-c(−RGDfK-)_4_ was performed (Figure 5A, B, D, E), and we also quantified the Hb content of the tumours after euthanasia (Figure 5C and 5F). No significant difference between mice injected with cancer cells alone or cancer cells and hMSCs was observed in either assay. We also found that the level of mouse CD31 mRNA expression was not affected by the presence of hMSCs (data not shown). We thus concluded that the treatment of the mice with hMSCs did not modify the overall amount of endothelial cells or the blood content of the tumours.

**Figure 6:**
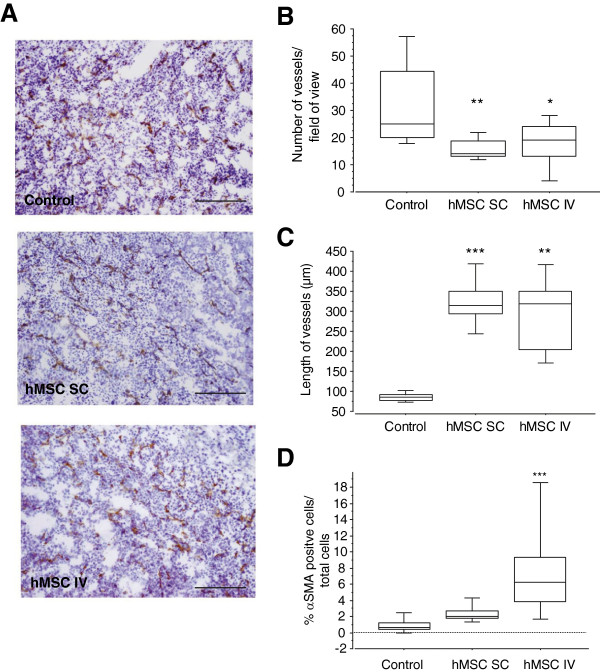
**hMSCs modify the SC tumour vessel structure. (A)** Images of vessels in TSA-pGL3 tumours in xenograft tumour models with and without injection (SC or IV) of hMSCs after staining with haematoxylin and a CD31 antibody (100× magnification). Scale bar = 200 μm. **(B** and **C)** Quantitative analyses of vessel density (B) and vessel length (C) The results are expressed as the mean ± SD. (n = 5 mice; six images/mouse; ^*^P <0.05, ^**^P = 0.0016, ^***^P <0.0001). **(D)** Box plots of αSMA^+^ cells in tumour. The results are expressed as the mean ± SD (n = 2 to 9 photographs/tumour; two to five mice/group; ^***^P <0.0001). hMSC, human mesenchymal stem cell; IV, intravenous; SC, subcutaneous; SD, standard deviation.

We then determined whether the vessel structure was different in the presence of hMSCs. Sections of subcutaneous tumours were stained with an antibody directed against CD31 to visualise the blood vessels (Figure 6A, B and C). Blood vessel density and blood vessel length were then quantified. The results of this analysis revealed that vessel area decreased in mice injected with TSA-pGL3 cells and hMSCs (SC (*P* = 0.0016) or IV (*P* = 0.0125)) compared with mice injected with tumour cells alone. We observed not only a decrease in the number of tumour vessels but also an increase in vessel length. Indeed, the median vessel length was 85 μm for tumour cells alone and 323 μm (*P* <0.0001) and 290 μm (*P* = 0.0016) when hMSCs were injected SC or IV. In addition, analyses of vessel maturation were performed by αSMA labelling (pericyte staining) in sections from subcutaneous TSA-pGL3 (control) and TSA-pGL3 + hMSCs tumours (see Figure 6D). In the control tumours, 1.06 ± 0.89% of cells were αSMA positive. The presence of hMSCs increased the percentages of αSMA-positive cells, to 2.32 ± 0.79% (*P* = 0.076) and 7.55 ± 4.51% (*P* <0.0001), when hMSCs were injected at the tumour periphery or IV, respectively.

Thus, the treatment of tumour-bearing animals with hMSCs does not modify the global number of CD31 and α_v_ß_3_-integrin positive tumour cells, nor does it change the blood content of the tumours, but it does affect the structure and shape of the blood vessels.

## Discussion

Numerous studies have clearly demonstrated that hMSCs can play an important role in cell therapy treatment against cancer. However, the reported results are highly controversial, and hMSC-based treatments are described as both promoting [10,11,21,36,37] and preventing tumour growth [8,38,39]. This dual effect can be observed in the same B16 melanoma model [22,40] and thus does not depend on tumour type. Some discrepancies could also be attributable to differences in the timing of hMSC administration (co-injection versus sequential injection), as suggested by Klopp *et al*. [34]. Indeed, when we subcutaneously co-injected hMSCs and tumour cells, we observed an increase of tumour growth compared to tumour cells alone (data not shown).

In this study, we demonstrated that the systemic or peritumoural injection of hMSCs resulted in a decrease in the pre-established tumour growth in mice using two different tumour models (TSA-pGL3 lung tumours and TSA-pGL3 subcutaneous tumours).

The effect of inhibition on tumour growth is clearly correlated with a decrease in the Ki67 labelling index. Our hypothesis is that this effect could be indirect because we observed the same result when hMSCs are injected by means of the IV route or directly beside the tumour site. Moreover, these results are obtained even when hMSCs are not or only poorly and transiently detected at the tumour site. In our model using nude mice, hMSCs were detected by RT-PCR one day after IV injection in the tumour as well as in the lung, liver and spleen, as previously described [41-44], but were undetectable when the mice were euthanized 7 days later. The tropism of hMSCs for tumours has been described in the literature [45-47] but remains marginal, with only 2% to 5% of injected cells found in the tumour masses [48]. It has been proposed that this tumour-growth inhibitory effect could be induced by hMSC-secreted soluble factors [9], which could act on tumour cells or on the tumour microenvironment. Prockop and colleagues proposed a model of myocardial infarction in which the action of hMSCs is indirect and related to inflammation. Moreover, the appearance of the hMSCs in the heart was transient and they disappeared by 48 hours [49,50].

Because different studies have demonstrated that MSCs play a role in angiogenesis, we decided to evaluate the influence of hMSCs on tumour angiogenesis. In a first assay, we used an *in vivo* physiological model of angiogenesis based on the introduction of a cellulose sponge template under the skin of the mouse and demonstrated the pro-angiogenic activities of hMSCs. Indeed, the *in vivo* angiogenic effect of hMSCs is as strong as the effect of basic FGF-2. This result was consistent with published data from another angiogenic, but less physiological, model of *in vivo* Matrigel implantation [14-16]. However, the pro-angiogenic activity of hMSCs was not confirmed in a pathological situation such as that in a tumour. Interestingly, the treatment of tumours with hMSCs was not associated with a modification of three-dimensional RGD-based fluorescence imaging or an increasing amount of haemoglobin. Nevertheless, we observed an effect on the tumour vasculature, characterised by a decrease in the number of blood vessels and an increase in the vessel size, leading to a more structured vascular architecture. The key role of hMSCs in regulating vessel maturation and functionality has already been described [51,52]. It is well known that tumours have an abnormal vascular network characterised by dilated, tortuous, and hyperpermeable vessels, and ultimately poor tumour oxygenation [53]. We also observed an increasing of the αSMA-positive cells in tumours after hMSC injection. It has been shown that pericyte maturation within tumours contributes to vascular normalisation [54]. In parallel, clinical evidence suggests that vascular normalisation occurs in human patients receiving anti-angiogenic agents [55] and that there is synergism between anti-VEGF therapy and systemic chemotherapy [56,57]. It will be interesting to test the injection of hMSCs associated with a conventional chemotherapy in our model. This approach has been evaluated by Pessina *et al*. [58] using MSCs as a vehicle for paclitaxel delivery. These authors observed an inhibition of endothelial cell proliferation *in vitro* and a diminution of B16 tumour growth but did not analyse the structure of the tumour blood vessels.

## Conclusions

In this study, we demonstrated that hMSCs administration decrease pre-established tumour growth, when hMSCs were injected systemically or directly into contact with the tumour. This effect is clearly correlated with an inhibition of tumour cell proliferation. More, whereas we have shown that the hMSCs induced angiogenesis *in vivo*, these cells do not alter the overall tumour angiogenesis. However, administration of hMSCs induces significant remodelling in tumour vasculature.

In summary, our results confirm that MSCs could be interesting to use for the treatment of pre-established tumours, particularly in combination with other therapies. However, the complex mechanisms of hMSC action remain to be characterised further.

## Abbreviations

FCS: Foetal calf serum; Hb: Haemoglobin; hMSC: Human mesenchymal stem cell; IV: Intravenous; PBS: Phosphate-buffered saline; RT-PCR: Reverse-transcriptase polymerase chain reaction; SC: Subcutaneous; SD: Standard deviation; TSA-pGL3: TS/A-pc mouse adenocarcinoma cell line stably transfected with the luciferase reporter gene.

## Competing interests

The authors declare that they have no competing interests.

## Authors’ contributions

MK, FDF, AK and CR were involved in the practical achievement of these experiments. Human stem cells for clinical use were obtained and characterized by AM, VP, FDF and MJR. Collection, analysis and interpretation of the data as well as the redaction were performed by CR, MK, FDF and JLC. Financial and administrative support was carried out by JLC and CR. All authors have read and approved the manuscript for publication.
